# Burden of Cardiovascular Disease among Multi-Racial and Ethnic Populations in the United States: an Update from the National Health Interview Surveys

**DOI:** 10.3389/fcvm.2014.00008

**Published:** 2014-11-10

**Authors:** Longjian Liu, Ana E. Núṅez, Yuan An, Hui Liu, Ming Chen, Jixiang Ma, Edgar Y. Chou, Zhengming Chen, Howard J. Eisen

**Affiliations:** ^1^Department of Epidemiology and Biostatistics, School of Public Health, Drexel University, Philadelphia, PA, USA; ^2^Department of Environmental and Occupational Health, School of Public Health, Drexel University, Philadelphia, PA, USA; ^3^Department of Medicine, Drexel University College of Medicine, Philadelphia, PA, USA; ^4^College of Computing and Informatics, Drexel University, Philadelphia, PA, USA; ^5^Department of Cardiology, First Affiliated Hospital of Chongqing Medical University, Chongqing, China; ^6^National Center for Chronic and Non-Communicable Disease Control and Prevention, Centers for Disease Control and Prevention, Beijing, China; ^7^Clinical Trial Service Unit & Epidemiological Studies Unit, Nuffield Department of Population Health, University of Oxford, Oxford, UK

**Keywords:** cardiovascular disease, risk factors, multi-race and ethnicity, USA

## Abstract

**Purpose:** The study aimed to provide new evidence of health disparities in cardiovascular disease (CVD) and diabetes mellitus (DM), and to examine their associations with lifestyle-related risk factors across the U.S. multi-racial and ethnic groups.

**Methods:** The analysis included a randomized population sample of 68,321 subjects aged ≥18 years old who participated in the U.S. 2012 and 2013 National Health Interview Surveys. Hypertension, coronary heart disease (CHD), stroke, and DM were classified according to participants’ self-report of physician diagnosis. Assessments of risk factors were measured using standard survey instruments. Associations of risk factors with hypertension, CHD, stroke, and DM were analyzed using univariable and multivariable analysis methods.

**Results:** Non-Hispanic (NH)-Blacks had significantly higher odds of hypertension, while Hispanics had significantly lower odds of hypertension, and NH-Asians and Hispanics had significantly lower odds of stroke than NH-Whites (*p* < 0.001). All minority groups, NH-Blacks, NH-Asians, and Hispanics had significantly higher odds of DM, but they had significantly lower odds of CHD than NH-Whites (*p* < 0.001). Increased body weight, cigarette smoking, and physical inactivity were significantly associated with increased odds of hypertension, CHD, stroke, and DM (*p* < 0.001). However, the strengths of associations between lifestyle-related factors and the study outcomes were different across racial and ethnic groups. NH-Asians with BMI ≥30 kg/m^2^ had the highest odds ratios (OR, 95% CI) for hypertension (5.37, 4.01–7.18), CHD (2.93, 1.90–4.52), and stroke (2.23, 1.08–4.61), and had the second highest odd ratios for DM (3.78, 2.68–5.35) than NH-Whites, NH-Blacks, and Hispanics.

**Conclusion:** CVD and DM disproportionately affect the U.S. multi-racial and ethnic population. Although lifestyle-related risk factors are significantly associated with increased odds of CVD and DM, the magnitudes of these associations are different by race and ethnicity.

## Introduction

Although since 1950, the incidence, prevalence, and mortality from cardiovascular diseases (CVD) have declined, coronary heart disease (CHD) and stroke (the two major forms of CVD) remain the leading causes of deaths in the United States of America (U.S.) (1–3). In 2010, 379,559 Americans died of CHD; it alone caused about one of every six deaths in the U.S. Approximately, every 1 min and 23 s, an American will die of CHD. Stroke caused about 1 of every 19 deaths. On average, every 40 s, someone in the U.S. has a stroke, and someone dies of one approximately every 4 min (1). In the U.S. more than 33%, or more than 100 million persons, identified themselves as belonging to a racial or ethnic minority population. Furthermore, it is well-known that CVD disproportionately affect the U.S. minority populations. However, limited studies address and provide updated evidence of the burden of CVD and its association with preventable risk factors across sub-groups of minority populations. In the present study, we aimed to examine the burden and health disparity of CVD and diabetes mellitus (DM), and their associations with lifestyle-related factors across multi-racial and ethnic groups in the U.S. We hypothesized that there were significant differences in the prevalence of CVD and DM and in the magnitude of the associations between CVD, DM, and risk factors across racial and ethnic groups. In the study, we used data from the most recent studies of the 2012 and 2013 National Health Interview surveys (NHIS). Findings from the study may provide significant evidence to health policy makers and moving forward prevention strategy and practice toward a significant reduction of cardiovascular disparity in the nation.

## Materials and Methods

Participants aged 18 years and older in the 2012 and 2013, NHIS were included in the study. The NHIS, a cross-sectional study, is an annual in-person interview-administered survey of health status and behaviors among the U.S. non-institutionalized population. One adult per household was chosen randomly to participate (4). We combined recently released 2012 and 2013 NHIS data in order to ensure the study sample size and statistical power were big enough when testing differences in cardiovascular health by racial/ethnic groups, Non-Hispanic (NH) Whites, NH-Blacks, NH-Asians, Hispanics, as well as subgroups of NH-Asians (i.e., Chinese, Indian, and Filipino groups). We examine the subgroups of NH-Asians because they are one of the fastest-growing racial/ethnic groups in the U.S. (5). Of 69,082 total participants aged 18 and older, we excluded 761 with missing values on race/ethnicity, yielding a final analytic sample of 68,321 adults. Data used in the study were de-identified and publicly available from the National Center for Health Statistics. Therefore, no specific institutional review board’s approval was needed (4).

This study focused on four major CVD related chronic conditions: hypertension, CHD, stroke, and DM. These conditions were defined on the basis of participants’ self-reports of diagnoses made by a doctor or health professional. Covariates included age, gender, education attainments ( <high-school graduate, high-school graduate, and ≥college), body mass index [BMI, calculated by weight (kg)/height (m^2^)], cigarette smoking (never smoked, formerly smoked, or currently smoke), alcohol consumption (non-drinker: <12 drinks in entire life, former drinker: no drinks in previous year, and current drinker), and physical activity. BMI was classified into four groups on the basis of the World Health Organization (WHO) definition (underweight: <18.5, normal weight: 18.5–24.9, overweight: 25–29.9, and obesity: ≥30 kg/m^2^). Physical activity status was grouped on the basis of current guidelines (active: ≥150 min per week of moderate-intensity equivalent leisure-time aerobic activity; insufficiently active: 10–149 min per week of moderate-intensity equivalent leisure-time aerobic activity) (6–8).

### Statistical analysis

A serial analysis was conducted to test the study hypotheses. The first group analysis included the basic characteristics description of the study participants by race/ethnicity, and tested difference using Chi-square tests. The second group analysis involved examination of associations of education, BMI, smoking, alcohol consumption, and physical activity status with prevalence of HBP, CHD, stroke, and DM by racial/ethnic groups. Further, the third group analysis looked at the racial/ethnic differences of the odds ratios (i.e., relative risks) for hypertension, CHD, stroke, and DM, using NH-White as the comparison group. Moreover, in the analysis, four multivariate logistic models were performed. Model 1 adjusted for age and sex. Model 2 adjusted for age, sex, and education. Model 3 adjusted for the covariates used in Model 2 plus three behavioral factors (smoking, alcohol consumption, and physical activity). Model 4 adjusted for the covariates used in Model 3 plus obesity. Similar to this analysis, we examined odds of hypertension, CHD, stroke, and DM across sub-groups of NH-Asians (American Chinese, Indians, and Filipinos) as compared to NH-Whites. Finally, we repeated the analysis by examining odds of hypertension, CHD, stroke, and DM across NH-Asian groups (Chinese, Indians, Filipinos, and other Asian groups including Japanese, Korean, and Vietnamese, with Chinese as the comparison).

All data analyses were performed using SAS version 9.3, with complex sample modules that take the sample design of NHIS, including stratification, clustering, and weight into consideration (SAS Institute, Cary, NC, USA). Statistical significance was determined for a two-sided test at a *p*-value <0.05.

## Results

### Characteristics of participants across racial/ethnic groups

Table 1 shows that of 68,321 participants in 2012 and 2013 NHIS, NH-Whites had a significantly higher mean age (50.74 years), followed by NH-Blacks (45.99 years), NH-Asians (43.74 years), and Hispanics (42.39 years). NH-Asians had the lowest proportion of those who received less than high-school education (6.82%), followed by NH-Whites (7.42%), NH-Blacks (13.91%), and Hispanics (30.56%) (*p* < 0.001). Similarly, lifestyle-related risk factors for CVD were significantly different across racial/ethnical groups. Overall, NH-Blacks had the worst CVD risk factor profiles (the highest obesity rate, higher current smoking rate, and the lowest physical activity rate) than the other racial/ethnic groups. Similar to this, NH-Blacks had the highest prevalence of hypertension (40.1%), stroke (3.9%), and DM (13.8%) than NH-Whites, NH-Asians, and Hispanics. However, NH-Whites had the highest prevalence of CHD (8.2%) among the four racial/ethnic groups.

**Table 1:** **Characteristics of participants by race/ethnicity**.

	NH-Whites (*n* = 41641)	NH-Blacks (*n* = 10600)	NH-Asians (*n* = 4278)	Hispanics (*n* = 11802)	p*
	% (SEP)	% (SEP)	% (SEP)	% (SEP)	
Age, years, mean (SEM)	50.74 (0.20)	45.99 (0.33)	43.74 (0.41)	42.39 (0.24)	0.0001
Male	46.08 (0.28)	40.92 (0.56)	46.55 (0.92)	46.83 (0.58)	<0.0001
Education					<0.0001
<High school	7.42 (0.19)	13.91 (0.48)	6.82 (0.46)	30.56 (0.65)	
High school	59.85 (0.38)	66.02 (0.58)	39.60 (1.10)	55.54 (0.62)	
≥College	32.73 (0.41)	20.06 (0.53)	53.58 (1.22)	13.91 (0.42)	
BMI, kg/m^2^					<0.0001
Under weight	1.89 (0.08)	1.27 (0.13)	4.23 (0.33)	1.16 (0.10)	
Normal weight	36.58 (0.32)	27.44 (0.56)	58.50 (0.93)	30.11 (0.50)	
Over weight	34.33 (0.30)	33.87 (0.49)	27.70 (0.82)	37.56 (0.52)	
Obesity	27.20 (0.29)	37.42 (0.58)	9.57 (0.56)	31.17 (0.53)	
Smoking					<0.0001
Never	53.40 (0.35)	65.62 (0.63)	77.09 (0.79)	71.44 (0.51)	
Former smoker	26.86 (0.30)	14.94 (0.44)	12.78 (0.60)	15.36 (0.38)	
Current smoker	19.74 (0.26)	19.44 (0.48)	10.13 (0.54)	13.20 (0.36)	
Alcohol consumption					<0.0001
Never	15.35 (0.28)	28.42 (0.57)	38.79 (0.96)	29.81 (0.56)	
Former drinking	15.66 (0.25)	16.67 (0.44)	9.18 (0.51)	13.26 (0.39)	
Current drinking	68.99 (0.36)	54.91 (0.66)	52.03 (0.90)	56.94 (0.61)	
Physical activity					<0.0001
Inactive	29.46 (0.45)	39.50 (0.64)	27.98 (1.07)	39.82 (0.68)	
Insufficiently active	30.36 (0.32)	28.31 (0.58)	32.97 (0.97)	25.10 (0.48)	
Active	40.18 (0.37)	32.19 (0.59)	39.06 (0.95)	35.08 (0.59)	
Chronic conditions					<0.0001
Hypertension	33.24 (0.32)	40.05 (0.73)	22.57 (0.81)	23.13 (0.47)	
CHD	8.20 (0.16)	6.76 (0.29)	3.78 (0.36)	4.65 (0.23)	<0.0001
Stroke	3.28 (0.10)	3.87 (0.20)	1.59 (0.23)	2.17 (0.16)	<0.0001
DM	9.86 (0.18)	13.79 (0.41)	8.48 (0.49)	10.99 (0.36)	<0.0001

*NH, non-Hispanic; SEM, standard error of mean; SEP, standard error of proportion; CHD, coronary heart disease; DM, diabetes mellitus*.

*p-Values for age-sex-adjusted differences across four race/ethnicity groups, except for tests of differences in age and sex among racial/ethnic groups.

### Odds of chronic conditions and associated risk factors by racial/ethnic groups

Table 2 shows that after adjustment for age and sex, subjects with lower education levels were significantly associated with elevated odds ratios for hypertension, CHD (Table 2A), stroke, and DM (Table 2B) in all racial/ethnic groups (*p* < 0.01). However, the strengths of these associations were much stronger in NH-Whites than the other race/ethnic groups (assessed by the values of odds ratios). NH-Asians with BMI ≥30 kg/m^2^ had the highest odds ratios for hypertension, CHD, and stroke, and had the second highest odd ratios for DM than the other racial/ethnic groups.

**Table 2:** **(A) Age-sex-adjusted odds ratios (95% CI) of education and other risk factors for hypertension and coronary heart disease by race/ethnicity and (B) Age-sex-adjusted odds ratios (95% CI) of education and other risk factors for stroke and diabetes by race/ethnicity**.

	NH-Whites		NH-Blacks		NH-Asians		Hispanics	
	OR (95% CI)	*p*	OR (95% CI)	*p*	OR (95% CI)	*p*	OR (95% CI)	*p*
**(A)**								
**Associated with odds of HBP**
Education
HS vs. ≥college	1.56 (1.47–1.65)	<0.0001	1.41 (1.22–1.61)	<0.0001	1.14 (0.95–1.37)	0.151	1.44 (1.22– 1.70)	<0.0001
<HS vs. ≥college	2.49 (2.26–2.74)	<0.0001	1.92 (1.61–2.29)	<0.0001	1.90 (1.46–2.48)	< 0.0001	1.75 (1.49– 2.07)	<0.0001
BMI Groups
Over weight vs. normal	1.75 (1.64–1.87)	<0.0001	1.64 (1.45–1.86)	<0.0001	1.99 (1.65–2.41)	< 0.0001	1.53 (1.34– 1.74)	<0.0001
Obesity vs. normal	3.43 (3.20–3.66)	<0.0001	3.41 (2.98–3.91)	<0.0001	5.37 (4.01–7.18)	< 0.0001	2.98 (2.58– 3.44)	<0.0001
Smoking status
Former vs. never	1.44 (1.36–1.53)	<0.0001	1.88 (1.64–2.16)	<0.0001	1.36 (1.07–1.73)	0.011	1.47 (1.29– 1.68)	<0.0001
Current vs. never	1.10 (1.02–1.18)	0.010	1.21 (1.08–1.37)	0.002	0.90 (0.65–1.26)	0.548	1.23 (1.03– 1.46)	0.019
Alcohol consumption
Former vs. never	1.13 (1.04–1.22)	0.003	1.69 (1.48–1.93)	<0.0001	1.31 (0.97–1.77)	0.083	1.30 (1.12– 1.51)	0.001
Current vs. never	0.72 (0.67–0.77)	<0.0001	1.08 (0.97–1.20)	0.180	0.72 (0.59–0.88)	0.002	0.80 (0.71– 0.91)	0.001
Physical activity
Insufficiently vs. active	1.24 (1.16–1.32)	<0.0001	1.20 (1.03–1.40)	0.019	1.05 (0.85–1.28)	0.668	1.15 (1.00– 1.32)	0.047
Inactive vs. active	1.64 (1.54–1.76)	<0.0001	1.37 (1.23–1.54)	<0.0001	1.08 (0.88–1.33)	0.458	1.24 (1.09– 1.42)	0.001
**Associated with odds of CHD**
Education
HS vs. ≥college	1.72 (1.54–1.93)	<0.0001	1.61 (1.20–2.15)	0.001	1.24 (0.86–1.79)	0.258	1.29 (0.90– 1.83)	0.162
<HS vs. ≥college	3.61 (3.11–4.19)	<0.0001	2.80 (2.07–3.78)	<0.0001	3.46 (2.29–5.22)	< 0.0001	2.14 (1.49– 3.10)	<0.0001
BMI Groups
Over weight vs. normal	1.23 (1.11–1.36)	<0.0001	0.99 (0.76–1.28)	0.921	1.46 (0.99–2.17)	0.057	1.01 (0.79– 1.28)	0.964
Obesity vs. normal	1.44 (1.29–1.61)	<0.0001	1.58 (1.27–1.96)	<0.0001	2.93 (1.90–4.52)	< 0.0001	1.37 (1.07– 1.74)	0.012
Smoking status
Former vs. never	1.84 (1.68–2.02)	<0.0001	1.91 (1.59–2.29)	<0.0001	1.90 (1.33–2.73)	0.001	1.72 (1.39– 2.13)	<0.0001
Current vs. never	1.54 (1.38–1.73)	<0.0001	1.33 (1.06–1.66)	0.013	1.00 (0.56–1.77)	0.994	1.27 (0.99– 1.63)	0.060
Alcohol consumption
Former vs. never	1.18 (1.04–1.35)	0.010	1.69 (1.31–2.16)	<0.0001	1.77 (1.15–2.74)	0.010	1.44 (1.11– 1.86)	0.007
Current vs. never	0.56 (0.50–0.63)	<0.0001	0.93 (0.74–1.16)	0.494	0.47 (0.32–0.69)	< 0.001	0.65 (0.52– 0.81)	<0.001
Physical activity
Insufficiently vs. active	1.18 (1.06–1.32)	0.003	1.79 (1.34–2.39)	<0.0001	1.11 (0.76–1.61)	0.586	0.97 (0.73– 1.29)	0.844
Inactive vs. active	2.02 (1.81–2.25)	<0.0001	2.40 (1.89–3.05)	<0.0001	1.57 (1.05–2.35)	0.029	1.66 (1.32– 2.10)	<0.0001
**(B)**								
**Associated with odds of stroke**
Education
HS vs. ≥college	1.84 (1.56–2.17)	<0.0001	2.00 (1.34–2.99)	0.001	1.64 (0.94–2.86)	0.083	1.46 (0.84– 2.53)	0.183
<HS vs. ≥college	3.98 (3.26–4.86)	<0.0001	2.77 (1.77–4.33)	<0.0001	2.63 (1.15–5.98)	0.022	2.67 (1.48– 4.81)	0.001
BMI Groups
Over weight vs. normal	1.16 (0.99–1.37)	0.068	0.97 (0.73–1.28)	0.819	1.44 (0.77–2.70)	0.260	0.74 (0.52– 1.05)	0.090
Obesity vs. normal	1.23 (1.04–1.45)	0.013	0.91 (0.70–1.19)	0.494	2.23 (1.08–4.61)	0.031	1.08 (0.74– 1.58)	0.696
Smoking status
Former vs. never	1.55 (1.36–1.77)	<0.0001	1.89 (1.49–2.39)	<0.0001	1.53 (0.86–2.73)	0.149	1.60 (1.12– 2.29)	0.011
Current vs. never	1.71 (1.45–2.01)	<0.0001	2.00 (1.56–2.55)	<0.0001	0.53 (0.23–1.25)	0.147	1.51 (0.95– 2.39)	0.084
Alcohol consumption
Former vs. never	1.10 (0.92–1.31)	0.298	1.54 (1.19–1.98)	0.001	1.32 (0.72–2.41)	0.368	1.76 (1.17– 2.66)	0.007
Current vs. never	0.43 (0.36–0.51)	<0.0001	0.77 (0.59–0.99)	0.045	0.23 (0.11–0.48)	< 0.0001	0.66 (0.46– 0.94)	0.021
Physical activity
Insufficiently vs. active	1.39 (1.15–1.67)	0.001	1.18 (0.81–1.72)	0.385	1.27 (0.57–2.84)	0.562	1.35 (0.90– 2.02)	0.145
Inactive vs. active	2.91 (2.45–3.45)	<0.0001	1.81 (1.29–2.52)	0.001	1.59 (0.76–3.32)	0.215	1.67 (1.18– 2.38)	0.004
**Associated with odds of DM**
Education
HS vs. ≥college	1.75 (1.59–1.92)	<0.0001	1.47 (1.22–1.77)	<0.0001	1.22 (0.96–1.55)	0.101	1.76 (1.40– 2.22)	<0.0001
<HS vs. ≥college	2.50 (2.17–2.87)	<0.0001	1.80 (1.43–2.27)	0.003	1.58 (1.13–2.22)	0.008	2.52 (2.02– 3.15)	<0.0001
BMI Groups
Over weight vs. normal	2.18 (1.95–2.43)	<0.0001	1.81 (1.49–2.20)	<0.0001	2.01 (1.53–2.65)	< 0.0001	1.73 (1.40– 2.14)	<0.0001
Obesity vs. normal	5.42 (4.86–6.04)	<0.0001	3.22 (2.68–3.87)	<0.0001	3.78 (2.68–5.35)	< 0.0001	3.34 (2.72– 4.11)	<0.0001
Smoking status
Former vs. never	1.26 (1.16–1.37)	<0.0001	1.64 (1.39–1.93)	<0.0001	1.20 (0.90–1.62)	0.217	1.66 (1.40– 1.96)	<0.0001
Current vs. never	0.99 (0.89–1.11)	0.844	0.95 (0.80–1.14)	0.610	0.87 (0.59–1.29)	0.501	1.09 (0.88– 1.37)	0.433
Alcohol consumption
Former vs. never	1.09 (0.98–1.21)	0.121	1.42 (1.17–1.72)	<0.001	1.20 (0.84–1.70)	0.316	1.35 (1.12– 1.64)	0.002
Current vs. never	0.51 (0.47–0.56)	<0.0001	0.71 (0.62–0.83)	<0.0001	0.63 (0.48–0.84)	0.001	0.68 (0.58– 0.81)	<0.0001
Physical activity
Insufficiently vs. active	1.49 (1.35–1.63)	<0.0001	1.23 (1.00–1.50)	0.047	1.52 (1.10–2.08)	0.010	1.12 (0.92– 1.37)	0.260
Inactive vs. active	1.99 (1.81–2.19)	<0.0001	1.45 (1.23–1.72)	<0.0001	1.94 (1.42–2.65)	< 0.0001	1.38 (1.17– 1.63)	<0.001

*NH, non-hispanic; HBP, hypertension; CHD, coronary heart disease; HS, high school; BMI, body mass index (kg/m^2^)*.

Subjects who were former smokers had significantly higher odds ratios for hypertension, CHD, stroke, and DM in NH-Whites, NH-Blacks, and Hispanics, and for hypertension and CHD in NH-Asians. Current smokers had significantly higher odds ratios for hypertension, CHD, and stroke in NH-Whites and NH-Blacks, and for hypertension in Hispanics, and borderline significance for CHD (*p* = 0.06) and stroke (*p* = 0.084) in Hispanics.

Former drinkers had higher odds ratios for hypertension, CHD, stroke, and DM across all racial/ethnic groups. However, current drinkers appeared to have an inverse association with hypertension, CHD, stroke, and DM in NH-Whites, NH-Asians, and Hispanics, but this inverse association was less consistent in NH-Blacks.

Physical inactivity was positively and significantly associated with odds of hypertension, CHD, stroke, and DM in NH-Whites, NH-Blacks, and Hispanics. This positive and significant association was observed only for CHD and DM in NH-Asians.

### Multivariate adjusted odds of cardiovascular conditions by racial/ethnic groups

### NH-Blacks, NH-Asians, and Hispanics vs. NH-Whites

**Figure 1:**
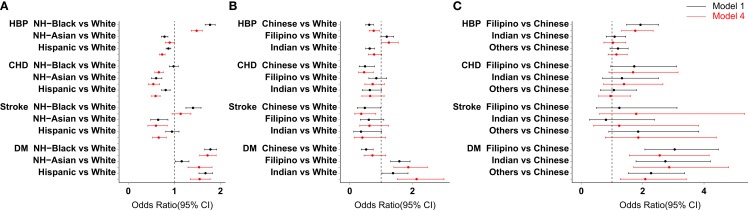
**Adjusted odds ratios (95% CI) of hypertension, CHD, stroke and DM for NH-Blacks, NH-Asians, and Hispanics vs. NH-Whites [(A), Models 1 and 4 in Table 3], Sub-groups of NH-Asians vs. NH-Whites [(B), Models 1 and 4 in Table 4], and Sub-groups of NH-Asians vs. Chinese Americans [(C), Models 1 and 4 in Table 5] – U.S. 2012 and 2013 National Health Interview Surveys**.

Table 3 and Figure 1A show that after adjustment for age, sex, education, smoking, alcohol consumption, physical activity status, and obesity (Model 4 in Table 3), NH-Blacks had significantly higher odds of hypertension (OR = 1.48, 95% CI: 1.37–1.61, *p* < 0.001) as compared to NH-Whites. However, Hispanics had significantly lower odds of hypertension than NH-Whites (OR = 0.74, 95% CI: 0.68–0.80, *p* < 0.001). NH-Blacks, NH-Asians, and Hispanics had significantly lower odds of CHD than NH-Whites. NH-Asians and Hispanics, except NH-Blacks had significantly lower odds of stroke than NH-Whites. It is of importance that all racial/ethnic minority groups (NH-Blacks, NH-Asians, and Hispanics) had significantly higher odds ratios for DM than NH-Whites (Figure 1A and Table 3).

**Table 3:** **Odds ratios (95% CI) of race/ethnicity differences (reference to NH-White) for hypertension, CHD, stroke, and DM**.

Covariates	NH-Whites (*n* = 41641)	NH-Blacks (*n* = 10600)		NH-Asians (*n* = 4278)		Hispanics (*n* = 11802)	
	Ref	OR (95% CI)	*p*	OR (95% CI)	*p*	OR (95% CI)	*p*
Hypertension
M1 age-sex-adjusted	1	1.77 (1.67–1.89)	<0.0001	0.79 (0.72– 0.86)	<0.0001	0.87 (0.82– 0.92)	<0.0001
M2 M1 + education	1	1.64 (1.55–1.74)	<0.0001	0.83 (0.76– 0.92)	<0.001	0.73 (0.68– 0.77)	<0.0001
M3 M2 + behavior factors	1	1.61 (1.51–1.71)	<0.0001	0.80 (0.72– 0.88)	<0.0001	0.72 (0.68– 0.77)	<0.0001
M4 M3 + Obesity	1	1.48 (1.37–1.61)	<0.0001	0.90 (0.81– 1.00)	0.056	0.74 (0.68– 0.80)	<0.0001
Coronary heart disease
M1 age-sex-adjusted	1	0.98 (0.89–1.08)	0.714	0.61 (0.50– 0.73)	<0.0001	0.81 (0.72– 0.91)	0.000
M2 M1 + education	1	0.86 (0.78–0.95)	0.003	0.65 (0.54– 0.78)	<0.0001	0.61 (0.54– 0.69)	<0.0001
M3 M2 + behavior factors	1	0.80 (0.72–0.90)	<0.0001	0.62 (0.51– 0.75)	<0.0001	0.62 (0.55– 0.71)	<0.0001
M4 M3 + Obesity	1	0.66 (0.58–0.76)	<0.0001	0.54 (0.44– 0.68)	<0.0001	0.59 (0.51– 0.69)	<0.0001
Stroke
M1 age-sex-adjusted	1	1.41 (1.25–1.58)	<0.0001	0.65 (0.49– 0.86)	0.003	0.95 (0.81– 1.10)	0.478
M2 M1 + education	1	1.21 (1.07–1.37)	0.002	0.70 (0.52– 0.93)	0.013	0.70 (0.60– 0.82)	<0.0001
M3 M2 + behavior factors	1	1.15 (1.01–1.32)	0.037	0.65 (0.48– 0.89)	0.006	0.72 (0.61– 0.85)	<0.0001
M4 M3 + Obesity	1	1.14 (0.95–1.36)	0.161	0.60 (0.43– 0.84)	0.003	0.66 (0.53– 0.83)	<0.001
Diabetes mellitus
M1 age-sex-adjusted	1	1.78 (1.66–1.92)	<0.0001	1.16 (1.02– 1.32)	0.023	1.68 (1.54– 1.83)	<0.0001
M2 M1 + education	1	1.63 (1.52–1.75)	<0.0001	1.23 (1.08– 1.40)	0.002	1.38 (1.26– 1.51)	<0.0001
M3 M2 + behavior factors	1	1.53 (1.42–1.65)	<0.0001	1.10 (0.96– 1.26)	0.152	1.35 (1.22– 1.48)	<0.0001
M4 M3 + Obesity	1	1.72 (1.55–1.91)	<0.0001	1.54 (1.30– 1.82)	<0.0001	1.55 (1.35– 1.78)	<0.0001

*NH, non-hispanic*.

### Sub-groups of NH-Asians compared to NH-Whites

Figure 1B and Table 4 show that among sub-groups of NH-Asians, Filipino Americans had higher odds of hypertension (OR = 1.25, 95% CI: 1.01–1.55, *p* = 0.044, Model 4) than NH-Whites. Indian Americans had significantly higher odds ratios for DM (OR = 2.13, 95% CI: 1.51–3.01, *p* < 0.001), followed by Filipino Americans (OR = 1.87, 95% CI; 1.41–2.48) as compared to NH-Whites. Chinese Americans had significantly lower odds of hypertension, CHD, and stroke as compared to NH-Whites.

**Table 4:** **Odds ratios (95% CI) of NH-Asians sub-groups (reference to NH-Whites) for hypertension, CHD, stroke, and DM**.

Adjusted covariates		NH-Asians					
	NH-Whites (*n* = 41641)	Chinese (*n* = 903)		Indian (*n* = 819)		Filipino (*n* = 931)	
	Ref	OR (95% CI)	*p*	OR (95% CI)	*p*	OR (95% CI)	*p*
Hypertension
M1 Age-sex-adjusted	1	0.62 (0.51–0.76)	<0.0001	0.64 (0.51– 0.79)	<0.0001	1.18 (0.99– 1.40)	0.060
M2 M1 + education	1	0.69 (0.57–0.84)	0.000	0.73 (0.58– 0.91)	0.006	1.22 (1.02– 1.46)	0.028
M3 M2 + behavior factors	1	0.64 (0.53–0.78)	<0.0001	0.65 (0.51– 0.83)	0.001	1.14 (0.94– 1.39)	0.171
M4 M3 + Obesity	1	0.77 (0.62–0.94)	0.013	0.78 (0.60– 1.01)	0.063	1.25 (1.01– 1.55)	0.044
Coronary heart disease
M1 Age-sex-adjusted	1	0.49 (0.30–0.79)	0.004	0.64 (0.40– 1.02)	0.060	0.85 (0.61– 1.18)	0.326
M2 M1 + education	1	0.54 (0.33–0.87)	0.012	0.72 (0.45– 1.15)	0.167	0.89 (0.64– 1.24)	0.496
M3 M2 + behavior factors	1	0.54 (0.34–0.85)	0.009	0.65 (0.40– 1.04)	0.074	0.82 (0.58– 1.16)	0.262
M4 M3 + Obesity	1	0.46 (0.28–0.75)	0.002	0.65 (0.39– 1.10)	0.108	0.73 (0.48– 1.11)	0.140
Stroke
M1 Age-sex-adjusted	1	0.48 (0.24–0.98)	0.042	0.36 (0.13– 1.02)	0.055	0.61 (0.34– 1.09)	0.094
M2 M1 + education	1	0.54 (0.26–1.10)	0.088	0.41 (0.14– 1.17)	0.095	0.65 (0.36– 1.16)	0.141
M3 M2 + behavior factors	1	0.52 (0.26–1.05)	0.067	0.35 (0.13– 1.00)	0.050	0.55 (0.28– 1.05)	0.068
M4 M3 + Obesity	1	0.37 (0.16–0.83)	0.017	0.40 (0.14– 1.14)	0.086	0.63 (0.32– 1.24)	0.179
Diabetes mellitus
M1 Age-sex-adjusted	1	0.52 (0.37–0.75)	0.000	1.38 (1.03– 1.86)	0.032	1.58 (1.30– 1.92)	<0.0001
M2 M1 + education	1	0.57 (0.38–0.84)	0.005	1.62 (1.20– 2.19)	0.002	1.68 (1.37– 2.05)	<0.0001
M3 M2 + behavior factors	1	0.49 (0.32–0.73)	0.001	1.32 (0.97– 1.79)	0.078	1.50 (1.21– 1.85)	0.000
M4 M3 + Obesity	1	0.72 (0.46–1.15)	0.168	2.13 (1.51– 3.01)	<0.0001	1.87 (1.41– 2.48)	<0.0001

*NH, non-hispanic*.

Figure 1C and Table 5 show that as compared to Chinese Americans, Filipino Americans had significantly higher odds ratios for hypertension (OR = 1.76, 95% CI: 1.32–2.34, Model 4). Filipinos, Indians, and other Asian groups (Japanese, Koreans, and all others) had significantly higher odds ratios for DM in the U.S., the corresponding odds ratios (95% CI) were 2.86 (1.71-4.80), 2.56 (1.57–4.17), and 2.09 (1.27–3.42), respectively.

**Table 5:** **Odds ratios (95% CI) of NH-Asian sub-groups (reference to Chinese) for hypertension, CHD, stroke, and DM**.

Adjusted covariates	Chinese (*n* = 907)	Indian (*n* = 819)		Filipino (*n* = 931)		Other Asian group (*n* = 1621)	
	Ref	OR (95% CI)	*p*	OR (95% CI)	*p*	OR (95% CI)	*p*
Hypertension
M1 Age-sex-adjusted	1	1.09 (0.81–1.45)	0.583	1.93 (1.48– 2.52)	<0.0001	1.20 (0.94– 1.53)	0.152
M2 M1 + education	1	1.08 (0.81–1.45)	0.609	1.87 (1.42– 2.45)	<0.0001	1.16 (0.91– 1.48)	0.221
M3 M2 + behavior factors	1	1.05 (0.78–1.41)	0.752	1.91 (1.46– 2.50)	<0.0001	1.18 (0.93– 1.51)	0.174
M4 M3 + obesity	1	1.03 (0.74–1.45)	0.856	1.76 (1.32– 2.34)	<0.001	1.14 (0.87– 1.50)	0.329
Coronary heart disease
M1 age-sex-adjusted	1	1.32 (0.70–2.52)	0.392	1.72 (0.96– 3.10)	0.069	1.06 (0.63– 1.80)	0.827
M2 M1 + education	1	1.33 (0.70–2.52)	0.393	1.69 (0.94– 3.03)	0.081	1.01 (0.60– 1.72)	0.957
M3 M2 + behavior factors	1	1.20 (0.64–2.22)	0.573	1.60 (0.90– 2.84)	0.108	0.93 (0.56– 1.56)	0.796
M4 M3 + obesity	1	1.39 (0.73–2.66)	0.318	1.69 (0.90– 3.15)	0.102	0.95 (0.57– 1.60)	0.850
Stroke
M1 age-sex-adjusted	1	0.80 (0.27–2.39)	0.695	1.24 (0.49– 3.12)	0.648	1.85 (0.90– 3.83)	0.096
M2 M1 + education	1	0.81 (0.27–2.41)	0.705	1.19 (0.47– 3.02)	0.707	1.76 (0.85– 3.64)	0.128
M3 M2 + behavior factors	1	0.72 (0.25–2.06)	0.541	1.18 (0.45– 3.08)	0.731	1.66 (0.81– 3.37)	0.165
M4 M3 + obesity	1	1.24 (0.40–3.82)	0.713	1.79 (0.60– 5.32)	0.295	1.86 (0.78– 4.40)	0.161
Diabetes mellitus
M1 age-sex-adjusted	1	2.74 (1.78–4.21)	<0.0001	3.04 (2.07– 4.47)	<0.0001	2.27 (1.54– 3.37)	<0.0001
M2 M1 + education	1	2.84 (1.80–4.49)	<0.0001	3.08 (2.04– 4.64)	<0.0001	2.28 (1.51– 3.46)	<0.001
M3 M2 + behavior factors	1	2.74 (1.72–4.35)	<0.0001	3.11 (1.99– 4.86)	<0.0001	2.32 (1.49– 3.60)	<0.001
M4 M3 + Obesity	1	2.86 (1.71–4.80)	<0.0001	2.56 (1.57– 4.17)	<0.001	2.09 (1.27– 3.42)	0.004

## Discussion

The present study, using data from nationally representative samples of the U.S. NHIS provides new evidence of cardiovascular epidemiology and heath disparity across the four largest racial/ethnic populations in the U.S. The results confirm that NH-Blacks had significantly higher odds of hypertension, and NH-Blacks, NH-Asians, and Hispanics had significantly higher odds of DM than NH-Whites. However, NH-Whites had significantly higher odd of CHD than NH-Blacks, NH-Asians, and Hispanics.

Beyond traditional thoughts that Asians including Asian Americans had lower risk of CVD as compared to the West, the present study is the first to examine and add new evidence to the body of literature regarding CVD risk, that of sub-groups of NH-Asians, Filipino Americans had significantly higher odds of hypertension than NH-Whites. Filipino and Indian Americans had significantly higher odds of DM than NH-Whites. These findings related to South Asians are consistent with our recent studies using data from the WHO coordinated World Health Surveys. A higher risk and prevalence of obesity and insulin resistance in South Asians might partially explain the racial and ethnic differences between South Asians and Whites (9). As there was no previous report that used a representative sample of Filipino Americans on the comparison of the study of interest between NH-Whites, and no data on serum and urine biomarker measures in the NHIS, we are unable to explain the racial and ethnic difference using the present data. Nevertheless, from prevention point of view, findings from the present study have an immediate impact on creating appropriate prevention strategies of CVD and DM across NH-Asian populations in the U.S.

In the study, we further observed that cigarette smoking appeared to have a lower impact (assessed by age-sex-adjusted odds ratios) on the risk of hypertension, CHD, stroke, and DM, but obesity appeared to have a higher impact on hypertension, CHD, and stroke in NH-Asians as compared to NH-Whites, NH-Blacks, and Hispanics. Although it is beyond the scope of further examination and discussion in the present study, the findings may emphasize that there are potentially different effects of different risk factors on the study outcomes by different race/ethnicity. It absolutely calls for further etiologic and mechanism studies.

As compared to former smokers, current smokers appeared to have lower odds of hypertension, CHD, stroke, and DM. It is likely due to the study data from cross-sectional designs that may cause “prevalence bias.” It may have occurred that patients with previous smoking status quit smoking after they were diagnosed with a disease (10). On alcohol consumption, former drinkers had a higher risk of hypertension, CHD, stroke, and DM. However, current drinking showed a protective effect on the study outcomes across all racial/ethnic groups. Although a possible “prevalence bias” may exist in this association as well, several studies have suggested there is a U-shaped association of alcohol consumption with CVD and DM (10–13).

The present study has several strengths. First, the results, on the basis of nationally representative datasets, provide new evidence of cardiovascular health and disparity across multi-racial/ethnic populations in the U.S. Although racial/ethnic disparities in CVD and DM have been reported in a number of studies, data from sub-groups of NH-Asian population is extremely limited. The current study fills in this gap by using a large-scale dataset from the NHIS. Finally, findings from the study not only add to new evidence of multiple minority cardiovascular health status, but also further address the importance of SES and lifestyle-related factors as predictors of cardiovascular health.

Similar to any study, the present study has several limitations. First, the findings are on the basis of data obtained from cross-sectional surveys, therefore, any cause-effect association between the predictors and outcomes cannot be interpreted (10). Second, the study outcomes were self-reports of physician-diagnosis of diseases (hypertension, CHD, stroke, and DM), information bias may occur. It should be noted that self-reports of physician-diagnosis of disease have been confirmed as a valid approach in population health surveys (14, 15). Third, the present study may have underestimated the prevalence of hypertension, CHD, stroke, and DM because no physical examination and measures of biomarkers from the blood sample were conducted. Last, but not least, the sample sizes of subgroups of NH-Asians are still small, as shown by a wider OR 95% CI (Figure 1C), further studies are needed by increasing the study sample sizes.

Despite these limitations, two clear and important conclusions follow the present study. First, CVD and DM disproportionately affect the U.S. multi-racial and ethnic population. Second, although lifestyle-related risk factors are significantly associated with increased odds of CVD and DM, the magnitudes of these factors on the study outcomes are different by racial and ethnic groups.

## Author Contributions

Longjian Liu and Ana E. Núṅez made the analysis design. Longjian Liu and Hui Liu did data analysis. Longjian Liu wrote the manuscript. Yuan An, Ming Chen, Jixiang Ma, Edgar Y. Chou, Zhengming Chen and Howard J. Eisen did critical review and gave valuable comments on this paper.

## Conflict of Interest Statement

The authors declare that the research was conducted in the absence of any commercial or financial relationships that could be construed as a potential conflict of interest.

## References

[1] Go AS Mozaffarian D Roger VL Benjamin EJ Berry JD Blaha MJ Heart disease and stroke statistics – 2014 update: a report from the American Heart Association. Circulation (2014) 129:e28–292. 10.1161/01.cir.0000442015.53336.12 24352519 PMC5408159

[2] Liu L Liu Z Ma M Xue F Sorel E The Cardiometabolic syndrome and risk of mortality from cardiovascular diseases and all causes among African Americans and White Americans. JCMD (2012) 3:1–9.

[3] Liu L Xue F Ma J Ma M Long Y Newschaffer CJ. Social position and chronic conditions across the life span and risk of stroke: a life course epidemiological analysis of 22 847 American adults in ages over 50. Int J Stroke (2013) 8(Suppl A100):50–55. 10.1111/j.1747-4949.2012.00927.x 23231424 PMC3610802

[4] NCHS. National Center for Health Statistics Data Sets. (2014). Available from: http://www.cdc.gov/nchs/index.htm

[5] Barnes PM Adams PF Powell-Griner E. Health characteristics of the Asian adult population: United States, 2004-2006. Adv Data (2008) 394:1–22. 18271366

[6] Li C Balluz LS Okoro CA Strine TW Lin JM Town M Surveillance of certain health behaviors and conditions among states and selected local areas – Behavioral Risk Factor Surveillance System, United States, 2009. MMWR Surveill Summ (2011) 60:1–250. 21849967

[7] Long Y Gracely EJ Newschaffer CJ Liu L. Analysis of the prevalence of cardiovascular disease and associated risk factors for European-American and African-American populations in the state of Pennsylvania 2005-2009. Am J Cardiol (2013) 111:68–72. 10.1016/j.amjcard.2012.08.045 23040600

[8] Liu L Nunez AE Yu X Yin X Eisen HJ for Urban Health Research Group. Multilevel and spatial-time trend analyses of the prevalence of hypertension in a large urban city in the USA. J Urban Health (2013) 90:1053–63. 10.1007/s11524-013-9815-x 23897041 PMC3853175

[9] Liu L Yin X Morrissey S. Global variability in diabetes mellitus and its association with body weight and primary healthcare support in 49 low- and middle-income developing countries. Diabet Med (2012) 29:995–1002. 10.1111/j.1464-5491.2011.03549.x 22150805

[10] Szklo M Nieto FJ Epidemiology Beyond the Basics. 2nd ed Sudbury, MA: Jones and Bartlett (2007).

[11] Hanna EZ Chou SP Grant BF. The relationship between drinking and heart disease morbidity in the United States: results from the National Health Interview Survey. Alcohol Clin Exp Res (1997) 21:111–8. 10.1097/00000374-199702000-00016 9046382

[12] Kim SY Breslow RA Ahn J Salem N Jr. Alcohol consumption and fatty acid intakes in the 2001-2002 National Health and Nutrition Examination Survey. Alcohol Clin Exp Res (2007) 31:1407–14. 10.1111/j.1530-0277.2007.00442.x 17561920

[13] Wannamethee SG Shaper AG. Lifelong teetotalers, ex-drinkers and drinkers: mortality and the incidence of major coronary heart disease events in middle-aged British men. Int J Epidemiol (1997) 26:523–31. 10.1093/ije/26.3.523 9222777

[14] Glymour MM Avendano M. Can self-reported strokes be used to study stroke incidence and risk factors? Evidence from the health and retirement study. Stroke (2009) 40:873–9. 10.1161/STROKEAHA.108.529479 19150869

[15] Bush TL Miller SR Golden AL Hale WE. Self-report and medical record report agreement of selected medical conditions in the elderly. Am J Public Health (1989) 79:1554–6. 10.2105/AJPH.79.11.1554 2817172 PMC1349815

